# miR-222-3p inhibits diabetic skin wound healing by targeting APLN

**DOI:** 10.3892/ijmm.2026.6002

**Published:** 2026-09-25

**Authors:** Ruyan Jie, Ying Tang, Tingting Wu, Chenfan Zhang, Man Song, Xiaotong Zhao, Mingwei Chen

**Affiliations:** 1 Department of Endocrinology, The First Affiliated Hospital of Anhui Medical University, Hefei, Anhui 230032, P.R. China; 2 The First Clinical Medical College, Anhui Medical University, Hefei, Anhui 230032, P.R. China; 3 Department of Endocrinology, The Shou County People's Hospital, Huainan, Anhui 232200, P.R. China

**Keywords:** miR-222-3p, apelin, diabetic foot ulcer, wound healing, methylglyoxal, angiogenesis

## Abstract

Diabetic foot ulcer (DFU) is a severe complication of diabetes, characterized by chronic, difficult-to-heal foot wounds. It has been hypothesized that miR-222-3p may influence wound healing via the regulation of angiogenesis. To elucidate the underlying mechanism, experimental investigations were conducted *in vitro* using human dermal microvascular endothelial cells (HMEC-1) and *in vivo* in a mouse model of streptozotocin-induced diabetes. Methylglyoxal (MGO), a glycolytic intermediate, is known to contribute to diabetic vascular complications. The results demonstrated that MGO-treated HMEC-1 cells exhibited an increased expression of miR-222-3p and impaired microvascular endothelial cell function. The overexpression of miR-222-3p in MGO-treated HMEC-1 cells further suppressed cell proliferation, migration and tube formation, while it enhanced apoptosis. Moreover, the upregulation of miR-222-3p reduced the expression of apelin (APLN), and dual-luciferase reporter assays confirmed APLN as a direct downstream target of miR-222-3p. The silencing of APLN markedly impaired HMEC-1 cell function. Notably, both the exogenous supplementation with Apelin-13 and the overexpression of APLN significantly reversed endothelial dysfunction caused by the upregulation of miR-222-3p. *In vivo*, in both normal and diabetic mice, the elevated expression of miR-222-3p in skin wounds reduced APLN levels in the peri-wound tissue and impaired wound healing. However, the overexpression of APLN or the inhibition of miR-222-3p with an antagomiR reversed this impairment. Collectively, these results suggest that miR-222-3p impairs microvascular endothelial cell function by targeting APLN, thereby contributing to delayed wound healing in diabetic skin.

## Introduction

Diabetic foot ulcer (DFU) is a prevalent chronic complication in patients with diabetes, characterized by difficulty in its treatment, a poor prognosis and a high recurrence rate. Individuals with diabetes with DFU are 2.5-fold more likely to succumb to the disease within 5 years compared to those without foot ulcers (1). Therefore, DFU should be considered as a critical warning sign of an increased risk of mortality in patients with diabetes. The pathogenesis of DFU is complex and remains incompletely understood. Current evidence suggests that peripheral neuropathy and peripheral vascular disease are the primary pathophysiological mechanisms driving the onset and progression of DFU (2). Wound healing generally occurs in three overlapping stages: The inflammatory phase, the neovascularization phase and the matrix remodeling phase. Disruptions at any stage can lead to delayed wound healing. This complex multistep mechanism entails the coordinated interplay among multiple cell types, notably comprising endothelial cells, keratinocytes, fibroblasts, along with diverse immune and inflammatory cell populations. Factors, such as hyperglycemia, persistent oxidative stress, inadequate angiogenesis and the dysfunction of immune-inflammatory cells at the wound site contribute to the difficulty in healing DFU wounds (3). Notably, reduced neovascularization, which leads to insufficient blood supply and impaired metabolism in the wound, is a key contributor to delayed skin wound healing in patients with diabetes (4).

MicroRNAs (miRNAs/miRs), functioning as endogenous non-coding regulatory molecules, mediate post-transcriptional gene silencing through sequence-specific interactions with complementary mRNA targets at 3'-untranslated regions, ultimately suppressing protein synthesis via translational repression or transcript destabilization mechanisms (5). miRNAs serve as critical regulators of cellular homeostasis, orchestrating fundamental biological programs through precise modulation of cell cycle progression, differentiation, apoptosis and immunological response. The aberrant expression of miRNAs can alter gene profiles, affecting a range of physiological processes and contributing to disease development (6). Increasing evidence indicates that the dysregulated expression of miRNAs is closely linked to the occurrence of DFU and impaired foot ulcer wound healing (7,8). Previous studies by the authors have identified that the altered expression of miR-204-3p, miR-34c and miR-155 plays a crucial role in the chronic wound healing of DFUs (9-11). Furthermore, a previous clinical study revealed that elevated levels of miR-222-3p in both peripheral blood and wound marginal tissues were closely associated with the onset of DFU and significantly affected its prognosis (12). Moreover, the expression of miR-222-3p in the peripheral circulating blood and peri-ulcer dermal specimens of patients with DFU demonstrated significant inverse associations with transcutaneous oxygen pressure, vascular endothelial growth factor (VEGF) and CD31 levels. These findings suggest that miR-222-3p may influence DFU wound healing through the regulation of angiogenesis, warranting further investigation.

Methylglyoxal (MGO) is an intermediate metabolite during glycolysis, typically detoxified via the glyoxalase system. However, in patients with diabetes, elevated glucose levels inhibit glyoxalase activity, leading to the accumulation of MGO in the body (13). Functioning as a principal pathogenic precursor to diabetic pathophysiology, MGO drives advanced glycation end-product formation, while perpetuating oxidative stress cascades, ultimately inducing the irreversible impairment of structural and functional integrity in critical macromolecules, including proteins, lipids and nucleic acids (14). It has been validated as a central mediator in the onset and progression of diabetic vascular complications (15-17). Berlanga *et al* (18) reported degenerative changes in the skin microvessels of rats chronically exposed to high MGO, including endothelial cell loss, thickening of the basement membrane, lumen occlusion and impaired acute granulation formation. These changes all led to vascular damage resembling that observed in diabetes (18).

Apelin (APLN) is a protein-coding gene predominantly expressed in endothelial cells of vascular tissues. The expression of APLN is associated with various physiological processes, such as heart development, fluid balance, angiogenesis and energy metabolism (19-22), and plays a role in the pathogenesis of conditions, such as heart failure, obesity, diabetes and cancer (23). Apelin, the peptide encoded by APLN, serves as a critical angiogenic factor that regulates endothelial cell migration, proliferation and apoptosis (24). Research has demonstrated that the apelin-APJ signaling pathway is essential for cardiovascular development, with the disruption of APLN expression in *Xenopus* embryos causing defects in vascular formation (25). In glioblastoma models, APLN knockdown has been shown to lead to reduced tumor vessel branching, decreased vessel density and impaired angiogenic budding (26). However, the role of APLN in neovascularization during DFU wound healing remains unexplored. In the ENCORI/starBase database (http://starbase.sysu.edu.cn/), potential binding sites between miR-222-3p and APLN were identified. Thus, the present study aimed to investigate whether miR-222-3p can modulate microvascular endothelial cell function by targeting APLN, thereby inhibiting wound healing in DFU.

## Materials and methods

### Cells and cell culture

Human dermal microvascular endothelial cells (HMEC-1) were obtained from the American Type Culture Collection (ATCC, CRL-3243). The cells were cultured in MCDB 131 complete medium (Gibco; Thermo Fisher Scientific, Inc.) supplemented with 10% fetal bovine serum (FBS; Nanjing Wisent Biotechnology Co., Ltd.), 10 mM L-glutamine (MedChemExpress), 10 ng/ml recombinant human epidermal growth factor (EGF; MilliporeSigma; Merck KGaA), 1 *μ*g/ml hydrocortisone (MilliporeSigma; Merck KGaA) and 1% penicillin-streptomycin (Beyotime Biotechnology). The cells were maintained at 37°C in a humidified incubator with 5% CO_2_. The medium was changed every 2-3 days, and cells were passaged upon reaching 80-90% confluency.

### Cell grouping and transfection

For transfection, cells in optimal growth conditions were used. miR-222-3p mimic (Guangzhou RiboBio Co., Ltd.) and si-APLN (Shanghai Sangon Biotech Co., Ltd.) were transfected using an *in vitro* miRNA/siRNA transfection reagent (Yisheng Biotechnology Co., Ltd.). Transfection of the APLN overexpression plasmid (Shanghai Sangon Biotech Co., Ltd.) was performed using Lipofectamine™ 2000 transfection reagent (Thermo Fisher Scientific Inc.). Following the manufacturer's instructions, transient transfection was performed when the cell confluency reached 50%. For each transfection, 2.5 *μ*g nucleic acid was used. The nucleic acid was incubated with the transfection reagent for 10 min at room temperature, and the transfection mixture was then added to the cells. Subsequent experiments were performed 48 h following transfection. HMEC-1 cells were randomly divided into the following groups: Normal control (NC), MGO intervention (MGO, MilliporeSigma; diluted with PBS to concentrations of 50, 100, 200, 400, 600, 800 and 1,000 *μ*mol/l), MGO group (400 *μ*mol/l), MGO + miR-con (MGO-treated HMEC-1 cells transfected with negative control miR-con), MGO + miR-222-3p (MGO-treated HMEC-1 cells transfected with miR-222-3p mimic), MGO + miR-222-3p + oe-APLN group (miR-222-3p mimic and APLN overexpression plasmid were co-transfected into MGO-treated HMEC-1 cells), MGO + miR-222-3p + Apelin-13 group [miR-222-3p mimic was transferred into MGO-treated HMEC-1 cells and 50 ng/ml Apelin-13 (MedChemExpress) was supplemented], MGO + si-APLN (MGO-treated HMEC-1 cells transfected with si-APLN), and MGO + si-NC (MGO-treated HMEC-1 cells transfected with negative control si-NC). The specific sequences of the miR-222-3p mimic and its negative control (miR-con) are proprietary to the supplier and are not disclosed. The siRNA sequences are listed in Table SI.

### Reverse transcription-quantitative polymerase chain reaction (RT-qPCR)

RT-qPCR was performed to measure the expression levels of miR-222-3p and APLN. Total RNA was isolated from cells, animal tissues and human tissues using TRIzol reagent (Thermo Fisher Scientific, Inc.). RNA concentration was quantified using a spectrophotometer (NanoDrop; Thermo Fisher Scientific, Inc.). According to the manufacturer's protocol, reverse transcription and quantitative PCR for miR-222-3p were performed using the miRNA reverse transcription and fluorescence quantitative detection kit (cat. no. E22003, Shanghai GenePharma Co., Ltd.). The PCR thermocycling conditions for miR-222-3p were as follows: An initial denaturation at 95°C for 3 min, followed by 40 cycles of 95°C for 12 sec and 62°C for 40 sec, with fluorescence signal acquisition at the 62°C step. Similarly, mRNA reverse transcription and amplification were conducted using the mRNA reverse transcription and fluorescence quantitative detection kit (cat. nos. 22107 and 22204, Shanghai Tolo Gang Biotechnology Co., Ltd.). The PCR thermocycling conditions for mRNA were as follows: An initial denaturation at 95°C for 30 sec, followed by 40 cycles of 95°C for 10 sec and 60°C for 30 sec, with fluorescence signal acquisition at the 60°C step. Relative expression levels of miR-222-3p and APLN were calculated using the comparative threshold cycle method (2^−ΔΔCq^) (27), implementing U6 snRNA and GAPDH as endogenous reference genes in a triplicate experimental design, and average values were used for analysis. The primer sequences are listed in Table SII.

### Western blot analysis

Total protein was extracted from cells, mouse wound tissues, and human tissue specimens using RIPA lysis buffer (Beyotime Biotechnology), with the subsequent quantification of protein concentrations conducted through bicinchoninic acid assay. Following denaturation by boiling, 15 *μ*g of protein samples were loaded into 12% SDS-PAGE gel wells for electrophoresis to separate proteins based on their molecular weight. The proteins were then transferred onto polyvinylidene fluoride (PVDF) microporous membranes. Following membrane transfer, the membranes underwent blocking in 5% (w/v) non-fat dairy protein solution (25°C, 2 h). The membranes were then incubated overnight (16-18 h) cold incubation (4°C) with primary antibodies diluted at pre-determined working concentrations, as follows: Rabbit anti-β-actin (1:10,000; cat. no. R380624, Chengdu Zen-Bioscience Co., Ltd.), rabbit anti-APLN (1:2,000; cat. no. BD-PT5163, Biodragon; https://www.biodragons.com/cn/goods/goodsView?GoodsId=23366&Catalog=), rabbit anti-IL-1β (1:1,000; cat. no. 16806-1-AP, Proteintech Group, Inc.), anti-IL-6 (1:1,000; cat. no. 21865-1-AP, Proteintech Group, Inc.) and anti-IL-8 (1:1,000; cat. no. 27095-1-AP, Proteintech Group, Inc.). Following overnight incubation (4°C), the membranes underwent sequential Tris-buffered saline-Tween 20 (TBST, Shanghai Sangon Biotech Co., Ltd., cat. no. B548105) rinses (3×8 min) prior to 2 h of exposure (25°C) to horseradish peroxidase-conjugated species-matched secondary antibodies (1:5,000; cat. no. 511203, Zen-Bioscience). The membranes were then rinsed with TBST three times again. Chemiluminescent signal acquisition was performed using a Tanon 5200 chemiluminescence imaging system (Tanon Science & Technology Co., Ltd.), followed by densitometric quantification via Image J software (version 1.8.0; National Institutes of Health) with β-actin normalization for relative protein expression determination.

### Cell viability assay

Cell viability was assessed using the Cell Counting kit-8 (CCK-8; Labgic Technology Co., Ltd.). Following cell digestion, cells from each group were resuspended, and the density was adjusted. Cells were plated at 5×10^3^/well density in 96-well microplates, with at least six replicates per group. Following the designated intervention, the CCK-8 reagent was diluted with serum-free medium at a 10:1 ratio to prepare the CCK-8 working solution, and 100 *μ*l of this solution were added to each well. Following 1 h of incubation under standard culture conditions (37°C, 5% CO_2_), optical density measurements were conducted at 450 nm using a multimode plate reader (Tecan Group Ltd.).

### Cell apoptosis assay

Cellular apoptosis was evaluated via dual-fluorochrome labeling using the Annexin V-FITC/PI Apoptosis Detection kit (cat. no. K2003, APExBIO Technology LLC) according to the manufacturer's protocols. Following designated intervention, the old culture medium was collected to terminate digestion. Adherent cells underwent enzymatic dissociation using EDTA-free trypsinization. The cell suspension was subjected to sequential centrifugation (300 × g, 5 min, 4°C) and dual PBS rinses at 4°C, followed by resuspension in 500 *μ*l binding buffer (cat. no. K2003, APExBIO Technology LLC). Fluorescent staining was achieved by the addition of 5 *μ*l Annexin V-FITC and 5 *μ*l propidium iodide (PI) working solutions, with subsequent light-protected incubation (25°C, 10 min). Immediate flow cytometric analysis was performed using the Beckman Coulter CytoFLEX LX platform.

### Cell scratch assay

The principle of the cell scratch assay is to evaluate coordinated cell migration driven by intercellular adhesion and matrix contact through the measurement of wound closure rates. It is primarily suitable for studies related to skin wound healing and epithelial repair (28-30). In the present study, when the cell density reached ~80-90%, two parallel lines were drawn on the bottom of the culture plate, followed by the application of two additional lines on the cell surface using a 200-*μ*l pipette tip, perpendicular to the parallel lines at the plate bottom. Post-PBS rinsing (2X), serum-depleted growth medium was introduced for controlled migration studies under standard incubation. The scratch wound was photographed at 0 and 24 h using the MShot digital imaging system (Guangzhou Mingmei Photoelectric Technology Co., Ltd.), with cell motility quantified as follows: Relative migration rate (%)=[(0 h scratch distance -24 h scratch distance)/0 h scratch distance] ×100%.

### Tube formation assay

In the tube formation assay protocol, 50 *μ*l Matrigel matrix was uniformly dispensed into 96-well culture plates and allowed to solidify at 37°C with 5% CO_2_ for 60 min. Following density calibration to 3×10^5^ cells/ml, 100 *μ*l of the standardized cell suspension were dispensed into each well using calibrated micropipettes. Following 4 h of incubation (37°C), tube formation was observed under the microscope and photographed using the MShot digital imaging system (Guangzhou Mingmei Photoelectric Technology Co., Ltd.). The total number of branch points and total tube length were quantified using ImageJ software (version 1.8.0; National Institutes of Health).

### Dual-luciferase reporter gene assay

The wild-type plasmid vector (APLN 3'-UTR-WT) and the mutant plasmid vector (APLN 3'-UTR-Mut) were constructed (General Bio Co., Ltd.). The two plasmids were co-transfected with either miR-222-3p mimic or miR-con using Lipo8000™ Transfection Reagent (cat. no. C0533, Beyotime Biotechnology) according to the manufacturer's instructions. For each transfection, 2.5 *μ*g nucleic acid was used. The transfection mixture was incubated at room temperature for 10 min and then added to the cells. The groups were classified as follows: miR-222-3p + APLN-WT, miR-con + APLN-WT, miR-222-3p + APLN-Mut, and miR-con + APLN-Mut. Following 48 h of transfection, 100 *μ*l Firefly luciferase detection reagent were added to each well according to the dual luciferase reporter gene assay kit protocol (cat. no. RG027, Beyotime Biotechnology). Firefly luciferase activity was measured using a multimode plate reader (Tecan Group Ltd.) after mixing. Subsequently, 100 *μ*l *Renilla* luciferase detection reagent (cat. no. RG027, Beyotime Biotechnology) were added, and *Renilla* luciferase activity was detected after mixing.

### Reactive oxygen species (ROS) detection

Intracellular ROS levels were measured using a ROS Assay kit (cat. no. S0033S Beyotime Biotechnology). Following washing with PBS, the cells were incubated with DCFH-DA working solution (included with the kit) diluted 1:1,000 in serum-free medium at 37°C for 30 min in the dark, with gentle mixing every 5-10 min. The cells were then washed three times with serum-free medium to remove extracellular probe. For nuclear staining, cells were incubated with Hoechst 33342 (cat. no. C1029, Beyotime Biotechnology; diluted 1:100 in serum-free medium) at 37°C for 15 min in the dark. Following incubation, the cells were washed three times with PBS. Fluorescence images were captured using a fluorescence microscope (Olympus Corporation; three random fields per group), and green fluorescence intensity indicated cellular ROS levels.

### Immunohistochemistry

Paraffin-embedded tissue sections from mouse peri-wound tissues (5-*μ*m thick) were routinely deparaffinized in xylene and rehydrated through graded ethanol. Heat-induced antigen retrieval was performed in citrate buffer (pH 6.0) under high pressure for 2 min. Endogenous peroxidase activity was blocked with 3% hydrogen peroxide at room temperature for 20 min. After washing with PBS, the sections were incubated with rabbit anti-CD31 antibody (cat. no. ab182981, 1:1,000, Abcam) and rabbit anti-VEGFA antibody (1:400; cat. no. GB15165, Wuhan Servicebio Technology Co., Ltd.) at 37°C for 60 min. Following washes with PBS, the sections were incubated with an HRP-conjugated goat anti-rabbit IgG secondary antibody (1:2,000; cat. no. ab205718, Abcam) at 37°C for 20 min. Following further PBS washes, immunoreactivity was visualized using 3,3'-diaminobenzidine (DAB; cat. no. ZLI-9018, Zsbio) substrate, and the sections were counterstained with hematoxylin (cat. no. BA-4041, Baso Diagnostics Inc.) at room temperature for 1 min, dehydrated through graded ethanol (cat. no. A500737, Shanghai Sangon Biotech Co., Ltd.), cleared in xylene (cat. no. A530011, Shanghai Sangon Biotech Co., Ltd.) and mounted with neutral resin (cat. no. C0173, Beyotime Biotechnology). Images were acquired using a light microscope (Nikon Corporation). Brown-yellow granular staining was considered positive.

### Clinical specimens

DFU tissues were obtained from patients with type 2 diabetes undergoing debridement or amputation (DFU group, n=12; 8 males and 4 females; age range, 50-78 years; median age, 61 years). Non-diabetic chronic lower limb ulcer tissues were collected from non-diabetic patients with chronic wounds as the controls (skin ulcer control, SUC group, n=12; 7 males and 5 females; age range, 45-72 years; median age, 58 years). All tissue specimens were collected at the First Affiliated Hospital of Anhui Medical University (Hefei, China) between June, 2023 and December, 2023. Written informed consent was obtained from all participants, and the study was approved by the Medical Ethics Committee of the First Affiliated Hospital of Anhui Medical University (Ethics batch number P20210039). Following surgical excision, tissue samples were immediately rinsed with sterile saline to remove blood, snap-frozen in liquid nitrogen, and stored at −80°C for RNA and protein extraction.

### Animal experiments

The study design involving animal models received ethical validation (approval no. LLSC20201040) from the Animal Care and Use Committee of Anhui Medical University (Hefei, China), with operational protocols strictly conforming to their guidelines. Male C57BL/6 mice (6-8 weeks old; median age, 7 weeks; n=70 males, 0 females; GemPharmatech Co., Ltd.) were intraperitoneally injected with streptozotocin (STZ, 50 mg/kg; cat. no. B2001, Beijing Boaigang Biotechnology Co., Ltd.) for 5 consecutive days to induce diabetes. Fasting blood glucose (FBG) levels were measured 2 weeks thereafter, and mice with FBG levels ≥16.7 mmol/l on two consecutive measurements were diagnosed as diabetic. Thereafter, FBG was monitored twice weekly to confirm persistent hyperglycemia. All diabetic mice were maintained in the hyperglycemic state for 4 weeks prior to use in subsequent experiments. Prior to wound creation, FBG and body weight were measured in all mice, and both parameters were monitored and recorded throughout the wound healing experiment (post-operative day 0 to day 8). The exclusion criteria were defined as follows: (i) FBG <16.7 mmol/l at any monitoring point during the experiment; (ii) >20% loss of initial body weight after wounding; (iii) severe health issues caused by hyperglycemia or infection, such as persistent lethargy, piloerection, or markedly reduced activity. Mice meeting any of these criteria were excluded from the final data analysis. No animals met these exclusion criteria during the experiment. For the APLN overexpression groups, AAV-oe-APLN vectors (1.0×10^11^ vector genomes/mouse) were administered by multi-point injection into the dorsal skin 3 weeks prior to wound creation. Both normal and diabetic mice were anesthetized with 1% sodium pentobarbital (50 mg/kg), and a full-thickness excisional wound (6 mm in diameter) was created on the dorsal midline. According to the experimental design, the following reagents were injected around the wound at a dose of 2.5 nmol per wound, every other day, for a total of four injections: miR-222-3p agomiR, agomiR negative control (agomiR-NC), miR-222-3p antagomiR and antagomiR negative control (antagomiR-NC) (Guangzhou RiboBio Co., Ltd.). The specific sequences of these products are proprietary to the supplier and are not disclosed. The groups were as follows: Ctrl + agomiR-NC, Ctrl + agomiR-222-3p, DM + agomiR-NC, DM + agomiR-222-3p, DM + agomiR-222-3p + oe-APLN, DM + antagomiR NC, and DM + antagomiR-222-3p (n=10 per group). On post-operative days 4 and 8, the mice were first deeply anesthetized with 1% sodium pentobarbital (50 mg/kg) administered intraperitoneally, and were then immediately euthanized by cervical dislocation. Death was confirmed by the cessation of respiration and bilateral pupillary dilation. Peri-wound tissues within ~2 mm of the wound margin were then collected. Of note, one portion of the tissue was snap-frozen in liquid nitrogen and stored at −80°C for RNA and protein analyses, and the remainder was fixed in 4% paraformaldehyde (cat. no. BL539A, Biosharp Life Sciences; at room temperature for 48 h), embedded in paraffin and processed for histological observation. The wound area was quantified using ImageJ software (version 1.8.0; National Institutes of Health), and the relative healing rate was calculated using the following formula: Relative healing rate (%)=[(day 0 wound area - day 8 wound area)/day 0 wound area] ×100%.

### Statistical analysis

All data were analyzed using GraphPad Prism 9.0 software. The normality of data distribution was assessed using the Shapiro-Wilk test. Normally distributed data are presented as the mean ± standard deviation (X̅ ± S). Comparisons between two groups were performed using an independent samples t-test, and comparisons among multiple groups were performed using one-way analysis of variance (ANOVA) followed by Tukey's post hoc test for pairwise comparisons. Non-normally distributed data are expressed as median (interquartile range) [M (P25, P75)] and were compared using the Mann-Whitney U test for two groups or the Kruskal-Wallis test with Dunn's multiple comparison test for multiple groups. All experiments were independently repeated at least three times. All statistical tests were two-sided, and P<0.05 was considered to indicate a statistically significant difference.

## Results

### MGO upregulates miR-222-3p expression in HMEC-1 cells

The glycolytic intermediate, MGO, is recognized as a contributor to vascular complications in diabetes. In a previous study, 400 *μ*mol/l MGO was employed to establish an *in vitro* diabetic wound healing model in human skin keratinocytes (31). In the present study, HMEC-1 cells were exposed to increasing concentrations of MGO (50, 100, 200, 400, 600, 800 and 1,000 *μ*mol/l). CCK-8 assay revealed that MGO inhibited cell proliferation in a concentration-dependent manner (Fig. 1A). At 400 *μ*mol/l, MGO induced a marked reduction in proliferation, while maintaining sufficient cell viability for subsequent functional assays. Thus, 400 *μ*mol/l MGO was used in subsequent experiments. To further validate the pathophysiological relevance of the selected MGO concentration, ROS production and pro-inflammatory cytokine expression in HMEC-1 cells treated with 400 *μ*mol/l MGO were assessed. The results revealed that intracellular ROS levels were significantly elevated in the MGO-treated group compared with the control group (Fig. 1B). Moreover, MGO treatment markedly upregulated the expression levels of IL-1β, IL-6 and IL-8 (Fig. 1C-E). These results demonstrated that 400 *μ*mol/l MGO effectively induced oxidative stress and inflammatory responses in HMEC-1 cells, supporting its suitability for use in subsequent experiments as a relevant pathophysiological stimulus. The present study then investigated whether MGO treatment affects miR-222-3p expression. RT-qPCR revealed that the miR-222-3p levels were significantly elevated in the MGO-treated cells compared with the untreated controls (Fig. 1F). This finding indicates that MGO-induced cellular stress is accompanied by the upregulation of miR-222-3p expression.

### miR-222-3p overexpression aggravates MGO-induced endothelial dysfunction

To further explore the role of miR-222-3p in MGO-treated HMEC-1 cells, we transfected a miR-222-3p mimic to overexpress miR-222-3p. Transfection efficiency was confirmed by RT-qPCR (Fig. 2A). miR-222-3p expression was significantly higher in the MGO + miR-222-3p group than in the MGO + miR-con group, confirming successful transfection. Subsequently, cell function was then evaluated by CCK-8 assay for cell proliferation, flow cytometry for apoptosis, scratch assay for migration ability, and tube formation assay for angiogenic capacity (Fig. 2B-E). Compared with the NC group, the MGO group exhibited a significantly reduced proliferation, increased apoptosis, impaired migration and diminished tube formation, as reflected by fewer total branch points and a shorter total tube length. Furthermore, compared with the MGO + miR-con group, the MGO + miR-222-3p group exhibited further reductions in proliferation, migration and tube formation, along with a further increase in apoptosis. These results indicate that the overexpression of miR-222-3p exacerbates MGO-induced injury in microvascular endothelial cells.

### miR-222-3p negatively regulates APLN

Through the analysis of the ENCORI/starBase database (http://starbase.sysu.edu.cn/), a nucleotide sequence within the 3'UTR of APLN mRNA with the potential to bind miR-222-3p was identified, suggesting APLN as a downstream target of miR-222-3p. To examine this, miR-222-3p was first overexpressed in HMEC-1 cells by transfection with a miR-222-3p mimic. The results of RT-qPCR and western blot analysis revealed that the overexpression of miR-222-3p significantly reduced both the APLN mRNA and protein levels compared with the miR-con group (Fig. 3A-C). Given that MGO treatment upregulated miR-222-3p expression, the present study then determined whether MGO can similarly suppress APLN expression. Indeed, in the MGO-treated HMEC-1 cells, both the APLN mRNA and protein levels were markedly decreased relative to the NC group (Fig. 3D-F), consistent with endogenous miR-222-3p upregulation under diabetic-like stress conditions. This inverse association was further validated in clinical specimens. The expression levels of miR-222-3p and APLN were simultaneously examined in tissues from patients with DFU and tissues from individuals with non-diabetic chronic lower limb ulcer (SUC). Compared with the SUC tissues, DFU tissues exhibited a significantly elevated expression of miR-222-3p and markedly reduced mRNA and protein levels of APLN (Fig. 3G-J), demonstrating a negative association between the two molecules in human diseased tissues. A dual-luciferase reporter assay was then performed to confirm the direct interaction. Co-transfection of the miR-222-3p mimic with the wild-type APLN 3'UTR reporter plasmid resulted in a marked decrease in luciferase activity compared with the miR-con group (Fig. 3K and L), whereas no significant difference in luciferase activity was observed between the two groups when the mutant plasmid was co-transfected. Collectively, these findings confirm that APLN is a direct downstream target of miR-222-3p, and this regulatory axis is conserved from *in vitro* MGO-induced stress to human DFU pathology.

### Silencing of APLN mimics miR-222-3p-induced endothelial dysfunction

Having confirmed that APLN is directly targeted and downregulated by miR-222-3p, the present study then wished to examine whether the loss of APLN itself is sufficient to drive the endothelial dysfunction caused by miR-222-3p. To this end, APLN was silenced in MGO-treated HMEC-1 cells using siRNA. RT-qPCR and western blot analysis were performed to assess the APLN mRNA and protein levels (Fig. 4A-C). Compared with the MGO + si-NC group, APLN mRNA and protein expression was significantly reduced in the MGO + si-APLN group, confirming the successful knockdown of APLN. Functional assays, including CCK-8 assay, flow cytometry, scratch assay and tube formation assay, were used to evaluate endothelial cell proliferation, apoptosis, migration and tube formation (Fig. 4D-G). In comparison with the MGO + si-NC group, the MGO + si-APLN group demonstrated a decline in cell proliferation activity, an elevation in the number of apoptotic cells, a reduction in cell migration capacity, and a decrease in both the total number of branch points and the total tube length of tubule formation. These data demonstrate that the silencing of APLN mimics the detrimental effects of miR-222-3p, reinforcing APLN as a critical functional mediator downstream of miR-222-3p.

### Restoration of APLN attenuates miR-222-3p-induced endothelial dysfunction

To determine whether APLN functionally mediates miR-222-3p-induced endothelial injury, rescue experiments were performed. Apelin-13, the most active subtype produced by the enzymatic cleavage of the APLN-encoded precursor protein, was used for exogenous supplementation. Previous studies have demonstrated that Apelin-13 activates the human APJ receptor with a half-maximal effective concentration (EC_50_) of ~0.3 nM and exerts concentration-dependent pro-proliferative, anti-apoptotic and endothelial protective effects within the 1-1,000-nM range (32-35). Accordingly, in the present study, a concentration of 50 ng/ml (~32 nM) Apelin-13 was selected, which far exceeds the receptor activation threshold, is sufficient to saturate the APJ receptor, and falls well within the established bioactive window. In parallel, APLN expression was restored at the genetic level by transfecting an APLN overexpression plasmid (oe-APLN). The successful overexpression was confirmed by RT-qPCR and western blot analysis, which revealed markedly elevated APLN mRNA and protein levels in the oe-APLN group relative to the control (Fig. 5A-C).

In the MGO-treated HMEC-1 cells, the overexpression of miR-222-3p significantly reduced cell proliferation, migration and tube formation, and markedly increased apoptosis compared with the control. Notably, both exogenous Apelin-13 supplementation and APLN overexpression significantly reversed these endothelial dysfunctions, as evidenced by restored proliferation, migration and tube formation, as well as reduced apoptosis (Fig. 5D-G). The highly consistent effects of the two rescue strategies collectively indicate that APLN is a critical downstream target required for miR-222-3p-mediated impairment of microvascular endothelial function.

### miR-222-3p impairs skin wound healing in mice

To further evaluate the impact of miR-222-3p on skin wound healing *in vivo*, both gain- and loss-of-function experiments were performed in normal and diabetic mice. miR-222-3p agomiR and its negative control (agomiR-NC) were injected locally into the peri-wound area of the dorsal skin in both diabetic and normal mice. The results revealed that, compared with the respective agomiR-NC groups, the miR-222-3p levels in the peri-wound skin were significantly elevated in the agomiR-222-3p groups of both diabetic and normal mice, while APLN expression was markedly reduced (Fig. 6A-D). Wound healing analysis demonstrated that, relative to their agomiR-NC controls, the agomiR-222-3p groups exhibited significantly delayed wound closure in both the diabetic and normal mice, indicating that miR-222-3p overexpression alone is sufficient to inhibit skin wound healing. To further determine whether this inhibitory effect is mediated by APLN downregulation, a rescue group in diabetic mice was established, in which APLN was overexpressed in the peri-wound skin via AAV vector prior to agomiR-222-3p treatment. The results revealed that APLN overexpression effectively reversed the delayed wound healing caused by agomiR-222-3p (Fig. 6E and F).

To assess whether silencing endogenous miR-222-3p promotes diabetic wound healing, DM + antagomiR-NC and DM + antagomiR-222-3p groups were established, in which miR-222-3p antagomiR was injected locally into the peri-wound area of diabetic mice. Compared with the antagomiR-NC group, the antagomiR-222-3p group exhibited significantly decreased peri-wound miR-222-3p levels, an elevated expression of APLN, and markedly accelerated wound closure, confirming the negative regulatory role of miR-222-3p in wound repair from the loss-of-function perspective (Fig. 6A-F).

To provide histopathological evidence for the underlying mechanisms, immunohistochemical staining for VEGFA and CD31 was performed on peri-wound tissues from all groups. In diabetic mice, the agomiR-222-3p group exhibited a reduced VEGFA and CD31 expression compared with the control group, whereas the antagomiR-222-3p group exhibited the opposite pattern. In normal mice, agomiR-222-3p similarly led to downregulation of VEGFA and CD31. Notably, in the rescue experiment, APLN overexpression significantly restored the VEGFA and CD31 levels, providing histopathological evidence that APLN is a key downstream effector through which miR-222-3p impairs wound angiogenesis (Fig. 7A-C). Taken together, these gain-and loss-of-function results demonstrate that miR-222-3p impairs skin wound healing *in vivo* by inhibiting angiogenesis.

## Discussion

MGO serves as a primary contributor to glycation and oxidative stress. Extensive research has demonstrated that the substantial accumulation of MGO plays a critical role in hindering wound healing and tissue repair in individuals with diabetes (36-39). Pang *et al* (40) demonstrated that the glyoxalase-mediated removal of MGO promoted the wound healing of DFUs by modulating the diabetic microenvironment and restoring the function of diabetic stem cells. MGO markedly decreases the protein expression of factors associated with angiogenesis, resulting in hindered neovascular formation. Conversely, the application of methylglyoxal scavengers improves MGO-induced endothelial cell dysfunction through the reduction of ROS generation, the suppression of cell apoptosis and the enhancement of angiogenic factor levels (41). Bone marrow-derived progenitor cells, crucial for vascular repair and angiogenesis, exhibit impaired tube formation, migration, proliferation and increased apoptosis when exposed to MGO (42). The present study confirmed that treatment of the HMEC-1 cells with MGO significantly elevated intracellular ROS levels and upregulated the expression of pro-inflammatory cytokines (IL-1β, IL-6 and IL-8), demonstrating the induction of glycative stress and inflammatory responses. Consistent with these findings, functional assays revealed that MGO exposure decreased cell proliferation, increased apoptosis, impaired migration and reduced tube formation, underscoring the detrimental role of MGO in endothelial injury and dysfunction. Notably, these phenotypic changes were accompanied by a marked upregulation of miR-222-3p, suggesting a potential involvement of miR-222-3p in mediating MGO-induced endothelial damage. However, the specific mechanism by which MGO regulates miR-222-3p expression warrants further investigation.

Insufficient angiogenesis and impaired blood supply due to endothelial cell dysfunction are major contributors to delayed wound healing. miR-222 is abundantly expressed in endothelial cells and plays a critical role in their function and regulation (43). Endothelial progenitor cells (EPCs) are crucial precursor cells that, under normal conditions, are rapidly mobilized from the bone marrow upon vascular injury and differentiate into mature vascular endothelial cells under the stimulation of various secreted cytokines, thereby playing a vital role in vascular repair and neovascularization. However, EPC function is often impaired in a high-glucose environment (44). A previous study indicated that the downregulation of miR-222-3p enhanced EPC function by activating the AMPK signaling pathway through targeting ADIPOR1, thereby promoting EPC migration, invasion and recruitment (45). miR-222-3p is highly expressed in endothelial cells of atherosclerotic vascular intima. Its upregulation inhibits PGC-1α protein production and reduces mitochondrial quantity, thereby inducing excessive ROS generation and apoptosis in human aortic endothelial cells, leading to endothelial cell injury and dysfunction (46). In the present study, HMEC-1 cells treated with MGO and subjected to miR-222-3p overexpression exhibited a further exacerbation in microvascular endothelial cell injury and dysfunction, as evidenced by further reductions in proliferation, migration and tube formation, and a further increase in apoptosis. Consistent with these *in vitro* findings, the local application of miR-222-3p agomiR significantly delayed wound healing in both normal and diabetic mice, whereas the silencing of endogenous miR-222-3p with antagomiR in diabetic wounds markedly accelerated wound closure. Histopathological analysis further revealed that miR-222-3p agomiR suppressed VEGFA and CD31 expression in peri-wound tissues, while antagomir treatment reversed these changes. Collectively, these results demonstrate that miR-222-3p functions as an anti-angiogenic miRNA that directly impairs microvascular endothelial cell function and inhibits skin wound healing.

miRNAs typically function by binding to the mRNA of downstream targets, inhibiting translation or promoting mRNA degradation (47). In the present study, miR-222-3p overexpression in HMEC-1 cells significantly suppressed APLN expression at both the mRNA and protein levels. Notably, MGO treatment similarly reduced APLN mRNA and protein expression, accompanied by a concomitant increase in endogenous miR-222-3p expression, indicating that this regulatory association is recapitulated under diabetic-like stress. Dual-luciferase reporter assays confirmed that APLN is a direct downstream target negatively regulated by miR-222-3p through sequence-specific binding to its 3'-UTR. *In vivo*, the elevated expression of miR-222-3p in the peri-wound tissue of diabetic mice was accompanied by the decreased expression of APLN. This inverse association was also observed in clinical specimens, where DFU tissues exhibited significantly elevated miR-222-3p levels and markedly reduced APLN expression levels compared with non-diabetic chronic ulcer tissues, providing translational relevance to the *in vitro* findings. Numerous studies have highlighted the importance of APLN in angiogenesis. Apelin signaling activates endothelial cells, driving them toward a highly migratory and proliferative state, thereby facilitating angiogenesis. Zebrafish lacking apelin signaling exhibit defects in endothelial tip cell morphology and sprouting (48). Moreover, apelin inhibits the NF-κB pathway and promotes endothelial cell proliferation upon binding to its receptor, alleviating hyperglycemia-induced microvascular dysfunction and inflammation. *In vivo* research has demonstrated that apelin enhances VEGFR2 expression, which promotes angiogenesis and increases microvessel density in the cardiac endothelial cells of diabetic mice (49). In the present study, the silencing of APLN expression in HMEC-1 cells through siRNA resulted in decreased proliferation and migration, increased apoptosis and reduced tube formation, indicating that the downregulation of APLN impairs endothelial cell function. Conversely, both exogenous Apelin-13 supplementation and APLN overexpression significantly rescued miR-222-3p-induced endothelial dysfunction *in vitro*. Notably, *in vivo* rescue experiments further demonstrated that APLN overexpression in the peri-wound skin effectively reversed agomiR-222-3p-induced delay in wound healing and restored angiogenesis markers in diabetic mice. Taken together, these findings establish a mechanistic cascade in which miR-222-3p directly targets APLN, suppresses its expression and thereby impairs microvascular endothelial function, ultimately contributing to delayed wound healing in diabetes.

The present study has several limitations which should be mentioned. First, although the Apelin-13 concentration used in the present study was selected based on published pharmacological evidence and falls within the established bioactive window, future dose-response studies will help to determine the optimal concentration for therapeutic application. Second, all *in vitro* experiments in the present study were performed using the human dermal microvascular endothelial cell line (HMEC-1). HMEC-1 is the first immortalized endothelial cell line successfully established from human dermal microvasculature, and it retains the morphological, phenotypic and functional characteristics of primary endothelial cells, including cobblestone morphology, the secretion of von Willebrand factor, the uptake of acetylated low-density lipoprotein, tube formation on Matrigel, and the expression of key markers, such as CD31, intercellular adhesion molecule-1 and vascular cell adhesion molecule-1 (50). This cell line has been extensively used and validated in studies on diabetic skin wound healing, angiogenesis, and diabetic complications (51-54). However, as an immortalized cell line, HMEC-1 may not fully recapitulate all physiological properties of primary human dermal microvascular endothelial cells. Therefore, while HMEC-1 provides a stable and reliable tool for mechanistic exploration in the present study, future investigations using primary cells are warranted to validate the key findings and further confirm the translational relevance of our conclusions.

Collectively, the *in vitro* and *in vivo* findings of the present study demonstrate that miR-222-3p impairs microvascular endothelial function and delays diabetic wound healing by directly targeting APLN. These results reveal a novel pathogenic mechanism in DFU and highlight the miR-222-3p/APLN axis as a potential therapeutic target for angiogenic therapies.

## Supplementary Data



## Figures and Tables

**Figure 1:**
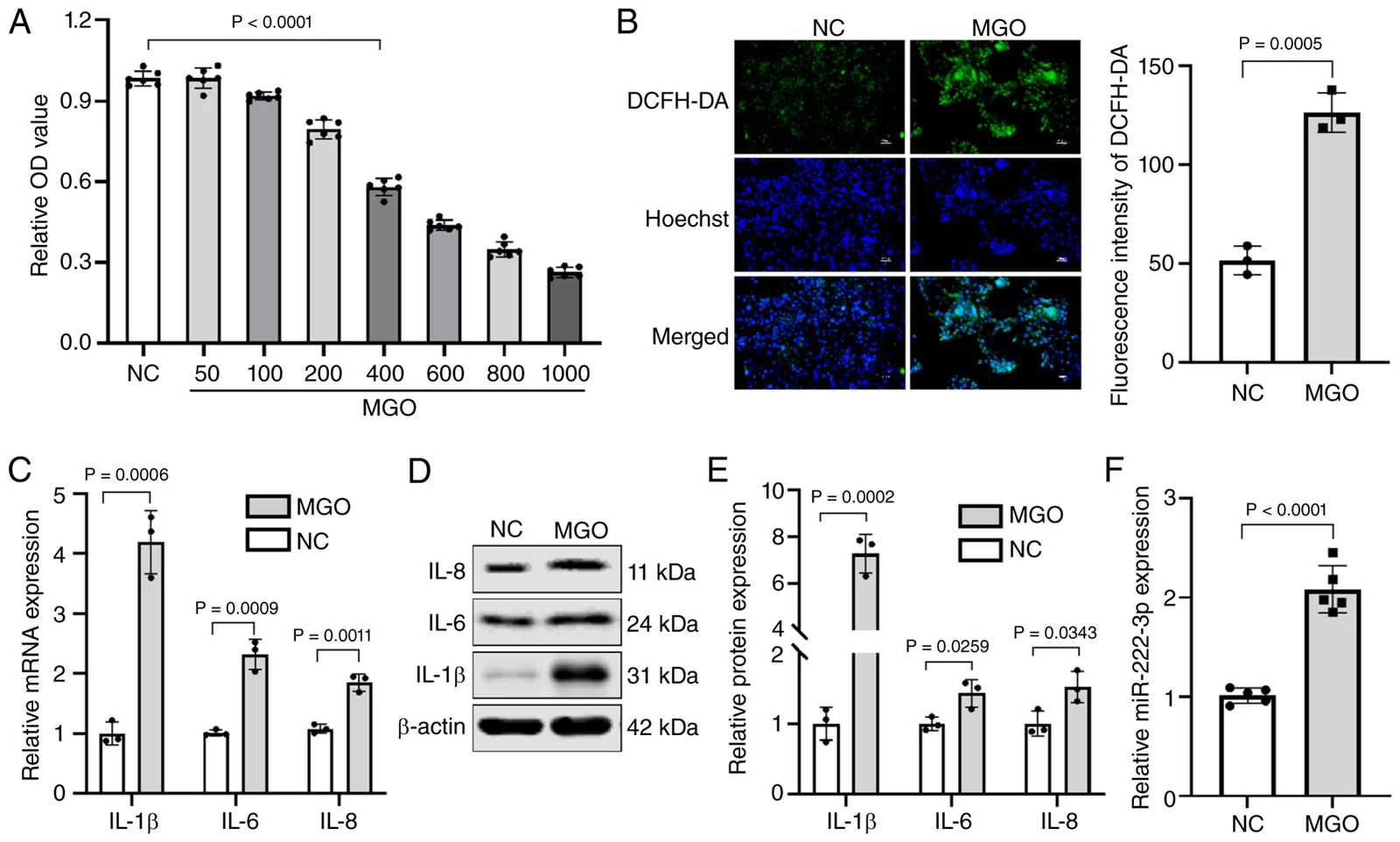
MGO upregulates miR-222-3p expression in HMEC-1 cells. (A) Cell proliferation in the NC group and groups treated with increasing concentrations of MGO was assessed using CCK-8 assay. (B) Reactive oxygen species detection in the NC and MGO groups. Scale bar, 200 *μ*m. (C) mRNA levels of inflammatory cytokines (IL-1β, IL-6 and IL-8) in the NC and MGO groups were measured by RT-qPCR. (D and E) Protein levels of IL-1β, IL-6 and IL-8 in the NC and MGO groups were detected using western blot analysis. (F) miR-222-3p expression in the NC and MGO groups was determined using RT-qPCR. All data are presented as the mean ± SD. Statistical analysis was carried out using an independent samples t-test or one-way ANOVA with Tukey's multiple comparisons test (n=3-6 biologically independent samples). NC, negative control; MGO, methylglyoxal; RT-qPCR, reverse transcription-quantitative PCR.

**Figure 2:**
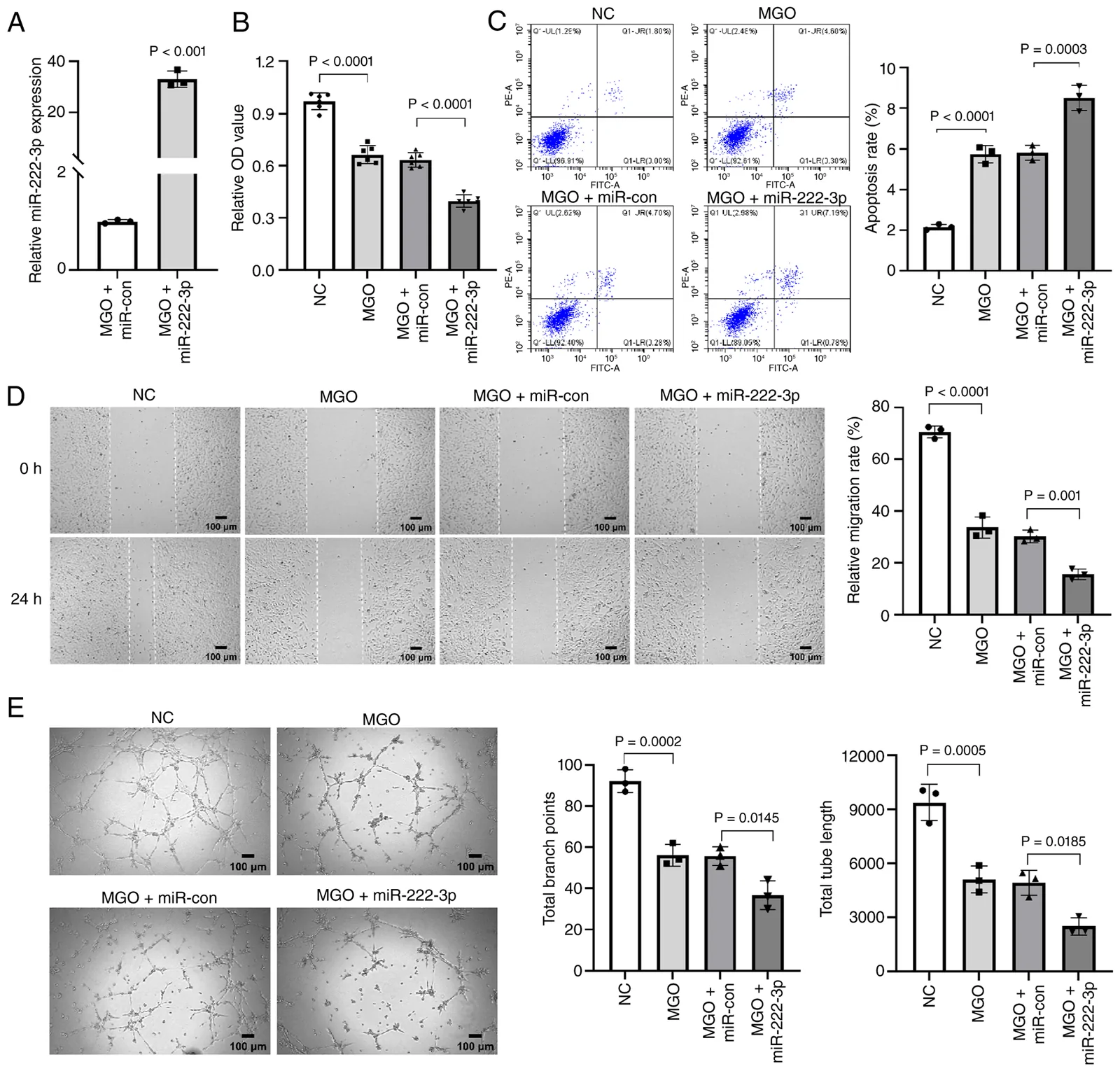
miR-222-3p overexpression aggravates MGO-induced endothelial dysfunction. (A) miR-222-3p expression levels in the MGO + miR-con and MGO + miR-222-3p groups were detected using reverse transcription-quantitative PCR. (B) Cell proliferation in the NC, MGO, MGO + miR-con and MGO + miR-222-3p groups was assessed using CCK-8 assay. (C) Apoptosis in the NC, MGO, MGO + miR-con, and MGO + miR-222-3p groups was detected using flow cytometry. (D) Cell migration in the NC, MGO, MGO + miR-con, and MGO + miR-222-3p groups was evaluated using scratch assay. Scale bars, 100 *μ*m. (E) Tube formation in the NC, MGO, MGO + miR-con, and MGO + miR-222-3p groups was assessed using tube formation assay. Scale bars, 100 *μ*m. All data are presented as the mean ± SD. Statistical analysis was carried out using an independent samples t-test or one-way ANOVA with Tukey's multiple comparisons test (n=3 or 6 biologically independent samples). NC, negative control; MGO, methylglyoxal.

**Figure 3:**
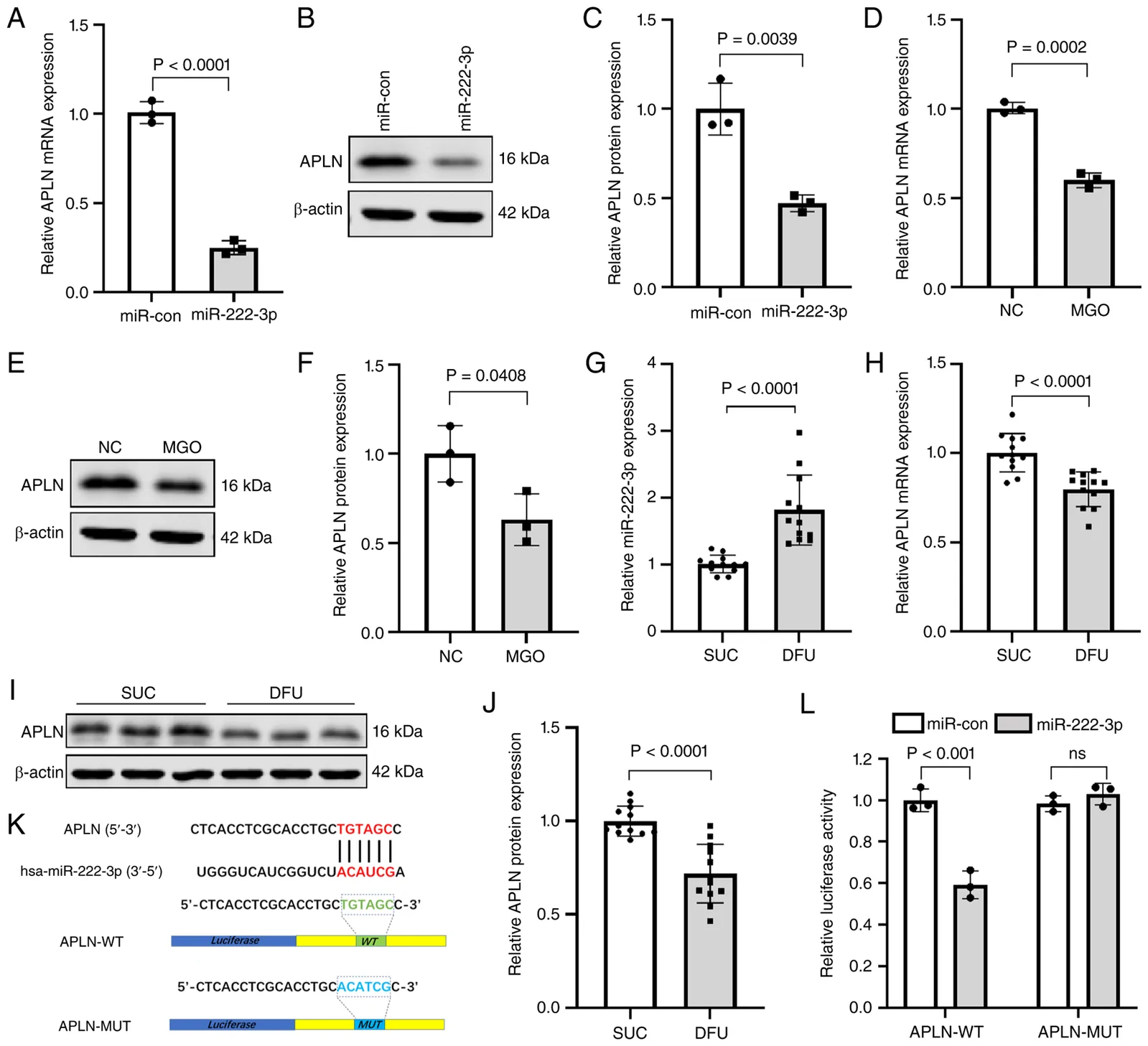
miR-222-3p negatively regulates APLN. (A) APLN mRNA expression in the miR-con and miR-222-3p groups was detected using RT-qPCR. (B and C) APLN protein expression in the miR-con and miR-222-3p groups was detected using western blot analysis. (D) APLN mRNA expression in the NC and MGO groups was detected using RT-qPCR. (E and F) APLN protein expression in the NC and MGO groups was detected using western blot analysis. (G) miR-222-3p expression in DFU tissues and SUC tissues was detected using RT-qPCR. (H) APLN mRNA expression in the DFU and SUC groups was detected using RT-qPCR. (I and J) APLN protein expression in the DFU and SUC groups was detected using western blot analysis. (K) Complementary nucleotide sequence between miR-222-3p and APLN. (L) Luciferase activity in the miR-con and miR-222-3p groups following co-transfection with wild-type (WT) or mutant (Mut) APLN 3'-UTR reporter plasmids. All data are presented as the mean ± SD. Statistical analysis was carried out using an independent samples t-test or one-way ANOVA with Tukey's multiple comparisons test (n=3 or 12 biologically independent samples). APLN, apelin; RT-qPCR, reverse transcription-quantitative PCR; NC, negative control; MGO, methylglyoxal; DFU, tissues from patients with diabetic foot ulcer; SUC, tissues from individuals with non-diabetic chronic lower limb ulcer.

**Figure 4:**
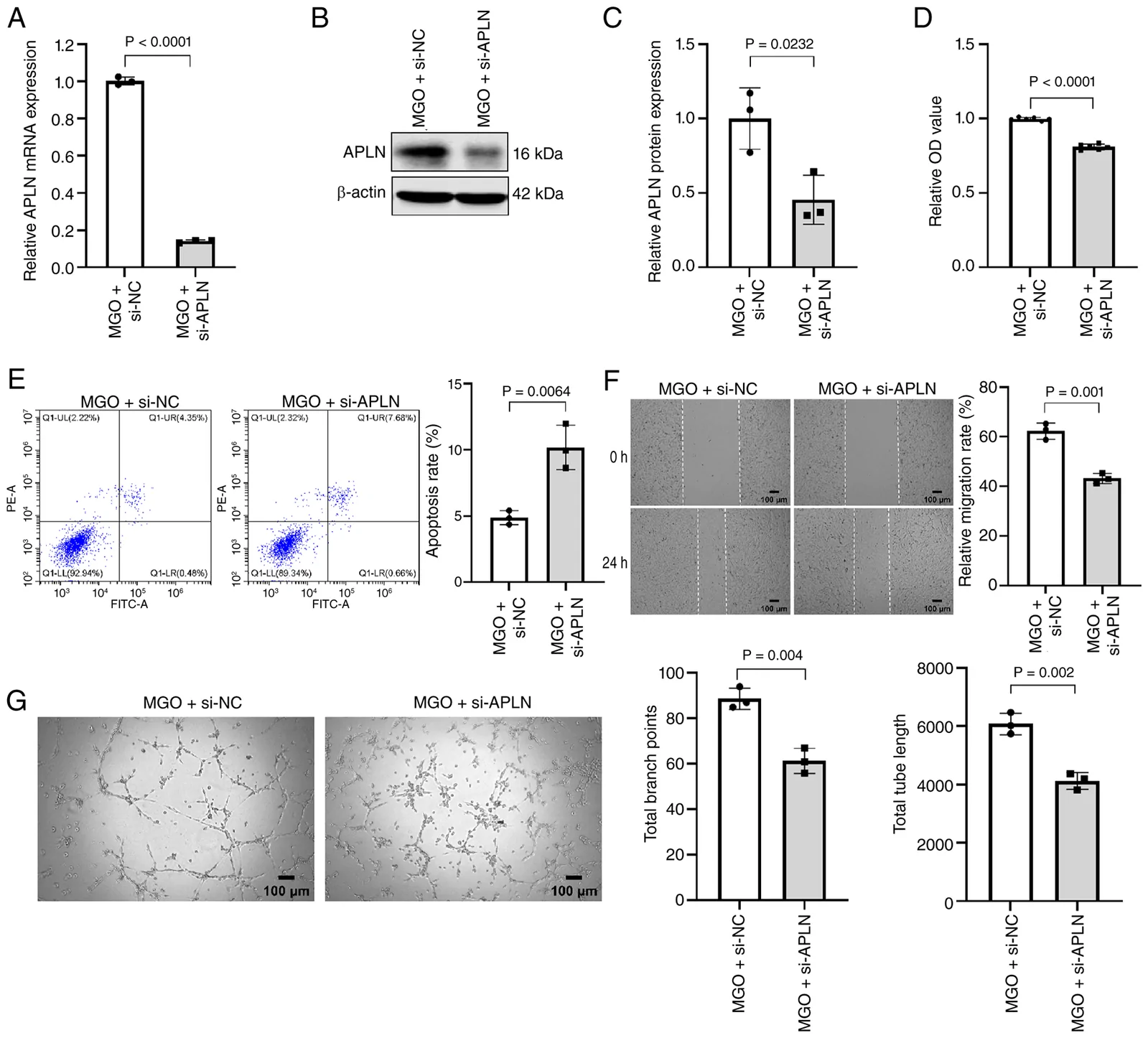
Silencing of APLN mimics miR-222-3p-induced endothelial dysfunction. (A) The mRNA expression of APLN in the MGO + si-NC and MGO + si-APLN groups was assessed using reverse transcription-quantitative PCR. (B and C) APLN protein expression in the MGO + si-NC and MGO + si-APLN groups was detected using western blot analysis. (D) The cell proliferative activity in the MGO + si-NC and MGO + si-APLN groups was measured using CCK-8 assay. (E) Apoptosis in the MGO + si-NC and MGO + si-APLN groups was detected using flow cytometry. (F) The migratory ability in the MGO + si-NC and MGO + si-APLN groups was evaluated using the scratch assay. Scale bars, 100 *μ*m. (G) Tube formation ability of the cells in the MGO + si-NC and MGO + si-APLN groups was assessed using tube formation assay. Scale bars, 100 *μ*m. All data are presented as the mean ± SD. Statistical analysis was carried out using an independent samples t-test or one-way ANOVA with Tukey's multiple comparisons test (n=3 biologically independent samples). APLN, apelin; NC, negative control; MGO, methylglyoxal.

**Figure 5:**
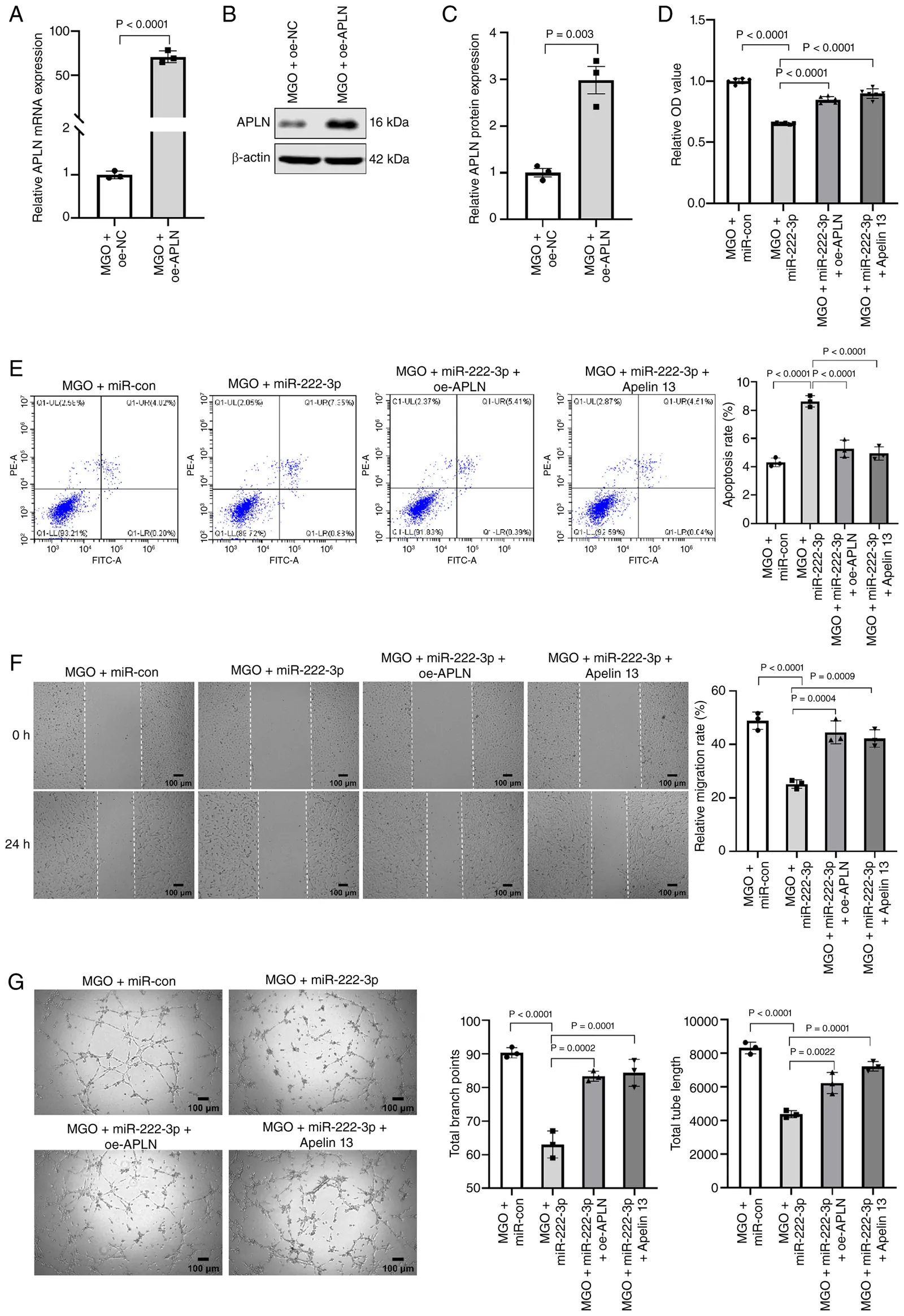
Restoration of APLN expression attenuates miR-222-3p-induced endothelial dysfunction. (A) The mRNA expression of APLN in the MGO + oe-NC and MGO + oe-APLN groups was assessed using reverse transcription-quantitative PCR. (B and C) APLN protein expression in the MGO + oe-NC and MGO + oe-APLN groups was detected using western blot analysis. (D) The cell proliferative activity in the MGO + miR-con, MGO + miR-222-3p, MGO + miR-222-3p + oe-APLN and MGO + miR-222-3p + Apelin-13 groups was measured using CCK-8 assay. (E) Apoptosis in the MGO + miR-con, MGO + miR-222-3p, MGO + miR-222-3p + oe-APLN and MGO + miR-222-3p + Apelin-13 groups was detected using flow cytometry. (F) Migration ability in the MGO + miR-con, MGO + miR-222-3p, MGO + miR-222-3p + oe-APLN and MGO + miR-222-3p + Apelin-13 groups was evaluated using the scratch assay. Scale bars, 100 *μ*m. (G) Tube formation ability of cells in the MGO + miR-con, MGO + miR-222-3p, MGO + miR-222-3p + oe-APLN and MGO + miR-222-3p + Apelin-13 groups was assessed using tube formation assay. Scale bars, 100 *μ*m. All data are presented as the mean ± SD. Statistical analysis was carried out using an independent samples t-test or one-way ANOVA with Tukey's multiple comparisons test (n=3 or 6 biologically independent samples). APLN, apelin; NC, negative control; MGO, methylglyoxal.

**Figure 6:**
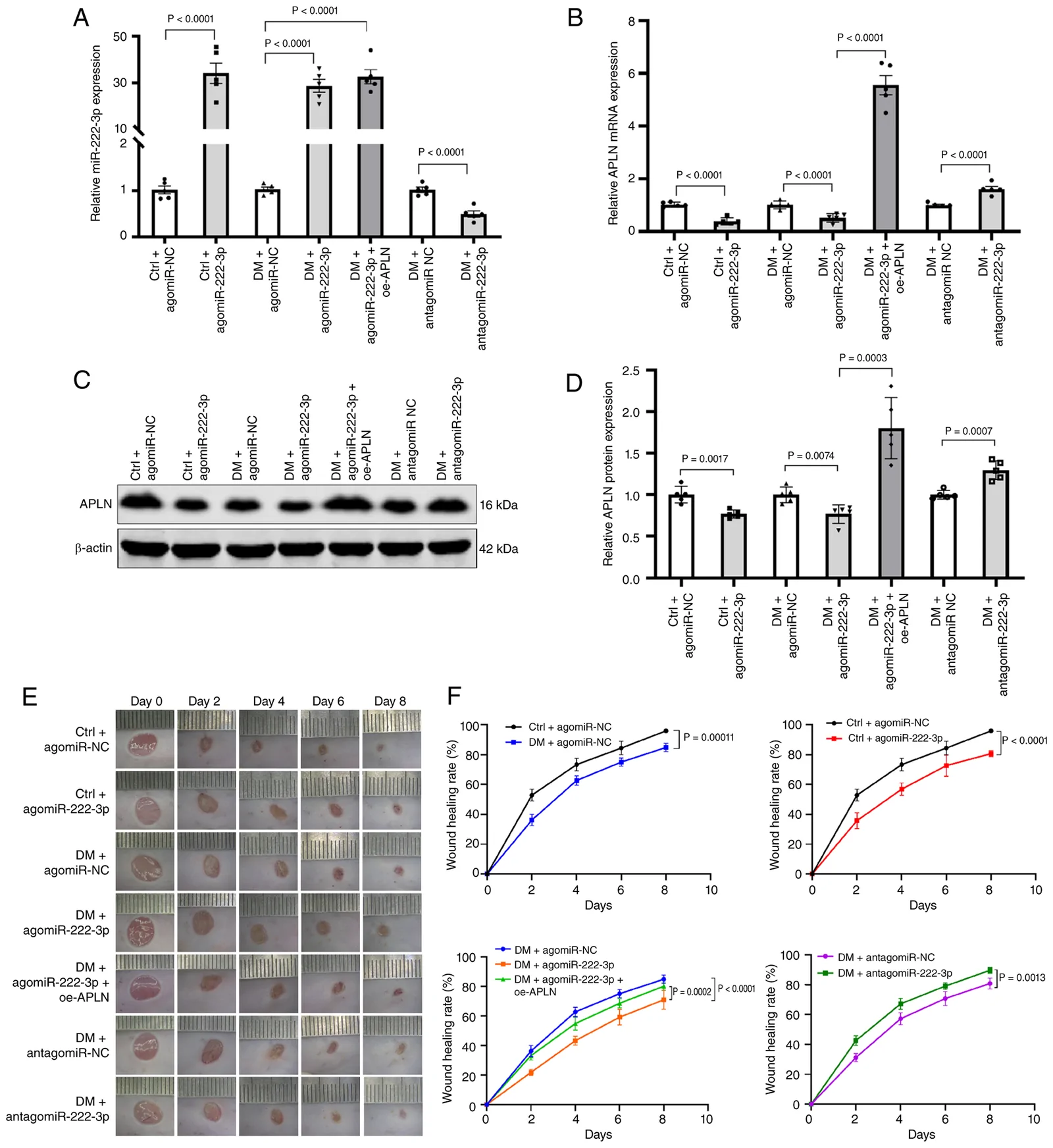
miR-222-3p impairs skin wound healing in mice. (A) The expression level of miR-222-3p in animal tissues was detected using RT-qPCR. (B) The mRNA expression level of APLN in animal tissues was detected using RT-qPCR. (C and D) The APLN protein expression level and its quantitative analysis were detected using western blot analysis and ImageJ software. (E and F) Representative images of the wound healing assay and the wound closure rate curve. All data are presented as the mean ± SD. Statistical analysis was carried out using an independent samples t-test or one-way ANOVA with Tukey's multiple comparisons test (n=5 biologically independent samples). RT-qPCR, reverse transcription-quantitative PCR; APLN, apelin; Ctrl, control; NC, negative control; DM, diabetes mellitus.

**Figure 7:**
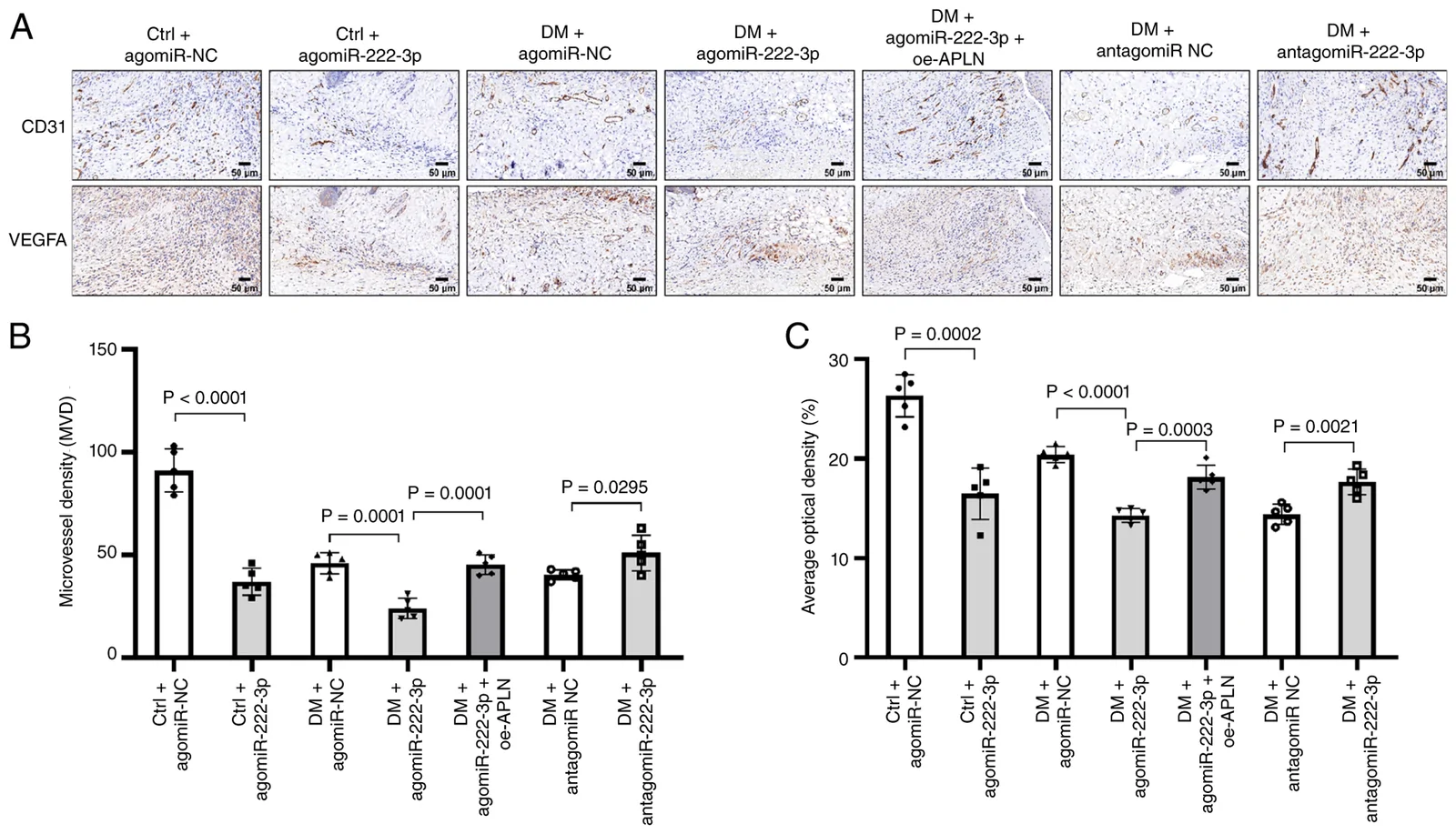
Immunohistochemical analysis of CD31 and VEGFA in wound tissues. (A) Representative immunohistochemical staining images of CD31 and VEGFA. Scale bars, 50 *μ*m. (B) Quantification of microvessel density. (C) Quantification of average optical density. All data are presented as the mean ± SD. Statistical analysis was carried out using an independent samples t-test or one-way ANOVA with Tukey's multiple comparisons test (n=5 biologically independent samples). CD31, cluster of differentiation 31, also known as platelet endothelial cell adhesion molecule-1, PECAM-1; VEGFA, vascular endothelial growth factor A; APLN, apelin; Ctrl, control; NC, negative control; DM, diabetes mellitus.

## Data Availability

The data generated in the present study may be requested from the corresponding author.
