# Supplementary material for: miR-222-3p inhibits diabetic skin wound healing by targeting APLN
Source: Int J Mol Med. 2026 Sep 25;58(5):331. doi: 10.3892/ijmm.2026.6002 (PMC13637565; doi:10.3892/ijmm.2026.6002)
Supplement: Supplementary file 1 [file Supplementary_Data.pdf]

Table SI. Sequences of the siRNAs used in the present study.

**Name**	**Sense (5′-3′)**	**Antisense (5′-3′)**
si-APLN	UGGGCUGGAAGACGGCAAUTT	AUUGCCGUCUUCCAGCCCATT
si-NC	UUCUCCGAACGUGUCACGU/dT//dT/	ACGUGACACGUUCGGAGAA/dT//dT/

APLN, apelin; NC, negative control.

Table SII. Specific primer sequences using RT-qPCR[a].

Gene	Forward sequence (5′-3′)	Reverse sequence (5′-3′)
(Human) miR-222-3p	GAAAGTTCGTCCAGCTACATCTG	TATGGTTGTTCTCGTCTCTGTGTC
(Human) U6	CAGCACATATACTAAAATTGGAACG	ACGAATTTGCGTGTCATCC
(Human) GAPDH	GGAAGGTGAAGGTCGGAGTC	AATGAAGGGGTCATTGATGG
(Human) APLN	GTCTCCTCCATAGATTGGTCTGC	GGAATCATCCAAACTACAGCCAG
(Human) IL-1β	CATGGGATAACGAGGCTTATGT	CATATGGACCAGACATCACCAA
(Human) IL-6	CACTCACCTCTTCAGAACGAAT	GCTGCTTTCACACATGTTACTC
(Human) IL-8	CTTGGCAGCCTTCCTGATTT	GGGTGGAAAGGTTTGGAGTATG
(Mouse) miR-222-3p	TGTTGCGAGCTACATCTGGC	TATGGTTCTTGACGACTGGTTGAC
(Mouse) U6	CAGCACATATACTAAAATTGGAACG	ACGAATTTGCGTGTCATCC
(Mouse) GAPDH	AGGTCGGTGTGAACGGATTTG	GGGGTCGTTGATGGCAACA
(Mouse) APLN	GTGGAGTGCCACTGATGTTG	CTGGTCCAGTCCTCGAAGTT

[a]For miR-222-3p detection, specific stem-loop RT primers were used. APLN, apelin.
